# Side-Chain Polarity Modulates the Intrinsic Conformational Landscape of Model Dipeptides

**DOI:** 10.1021/acs.jpcb.1c02412

**Published:** 2021-05-26

**Authors:** Debayan Chakraborty, Atreyee Banerjee, David J. Wales

**Affiliations:** † Department of Chemistry, The University of Texas at Austin, 24th Street Stop A5300, Austin, Texas 78712, United States; ‡ Yusuf Hamied Department of Chemistry, University of Cambridge, Lensfield Road, Cambridge CB2 1EW, United Kingdom; § Max Planck Institute for Polymer Research, 55128 Mainz, Germany

## Abstract

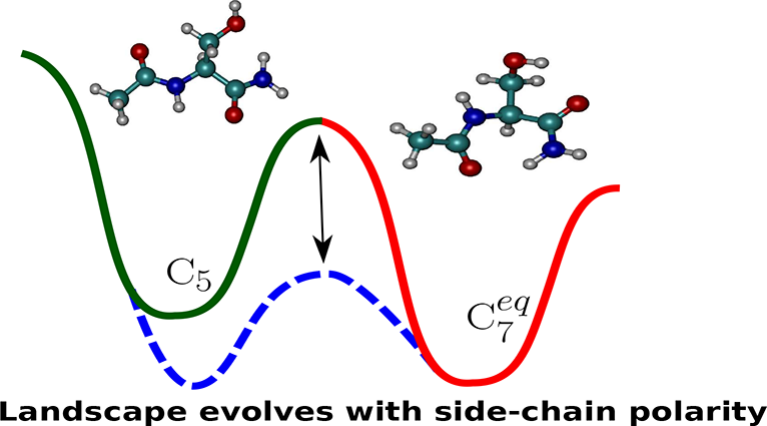

The intrinsic conformational preferences of small peptides may provide additional insight into the thermodynamics and kinetics of protein folding. In this study, we explore the underlying energy landscapes of two model peptides, namely, Ac-Ala-NH_2_ and Ac-Ser-NH_2_, using geometry-optimization-based tools developed within the context of energy landscape theory. We analyze not only how side-chain polarity influences the structural preferences of the dipeptides, but also other emergent properties of the landscape, including heat capacity profiles, and kinetics of conformational rearrangements. The contrasting topographies of the free energy landscape agree with recent results from Fourier transform microwave spectroscopy experiments, where Ac-Ala-NH_2_ was found to exist as a mixture of two conformers, while Ac-Ser-NH_2_ remained structurally locked, despite exhibiting an apparently rich conformational landscape.

## Introduction

Despite decades of research, the protein folding problem^1,2^ continues to attract great interest. The major advances in specialized hardware,^3^ as well as deep-learning techniques,^4^ now make it possible to study proteins of varying complexities and topologies using computer simulations and provide the much-needed atomistic insight into biophysical experiments.^5,6^ There are essentially two facets to the folding problem: the first one is related to structure prediction and thermodynamics and includes the characterization of the three-dimensional structures that dominate the equilibrium population. These structures are usually associated with specific biological functions within the cellular machinery. The second problem concerns folding mechanisms and kinetics, which describe how a protein relaxes to its functional three-dimensional conformation starting from a relatively unstructured configuration by navigating along well-defined folding routes on the underlying energy landscape.^7,8^ In addition to the intramolecular energetics, as dictated by the interactions among the constituent amino acids, intermolecular interactions with the surrounding environment also have a pronounced effect on the folding thermodynamics and kinetics. As shown in previous studies, solvent polarity, pH,^9−11^ as well as the presence of crowding agents,^12,13^ and denaturants,^14,15^ significantly reshape the folding landscape, thereby altering its emergent properties.

The synergy between intra- and intermolecular interactions exacerbates the complexity of the folding process, making it rather difficult to formulate general principles that would advance our understanding of the structure–function paradigm. A first step in this direction is to explore the intrinsic conformational preferences of protein sequences, particularly small peptides and peptidomimetics, in the absence of interactions with the surroundings. Experiments carried out in the gas-phase provide the ideal medium for such studies and help to discern the interplay of intramolecular interactions that lend stability to the protein molecule and dictate its three-dimensional structure.^16^ While double resonance experiments have long been exploited to investigate the structural features of small peptides,^17−23^ microwave spectroscopy has recently emerged as a potential alternative. In recent studies,^24−34^ Alonso and co-workers have determined the structures of the dominant conformers for a range of capped dipeptides, using a combination of the laser ablation technique and Fourier transform microwave spectroscopy. The authors conclusively show that the polarity of the side-chain plays a key role in dictating the conformational equilibrium of the dipeptides, through the locking of specific configurations.^29,30^ In addition to gas-phase experiments, a number of theoretical studies have characterized the potential energy landscape (PEL) for different dipeptides, using molecular mechanics as well as *ab initio* methods. Tobias and Brooks used the CHARMM force field to study the conformational equilibria of the alanine dipeptide, both in the gas-phase, and in water and observed a pronounced solvent effect.^35^ In another study, Vondrášek and co-workers^36^ used metadynamics simulations to explore the free energy landscape (FEL) of the alanine dipeptide in vacuum, as well as in water, for three different parametrizations of the AMBER force field. The populations of the different secondary structure elements predicted by the authors were found to be in reasonable agreement with those estimated using vibrational spectroscopy.^37^ Systematic benchmarking on model peptides carried out by Jensen and co-workers^38^ revealed that the commonly used biomolecular force fields based on fixed charged models could not reproduce the reference data from high-level quantum mechanical calculations, and multiple moments and polarizability needed to be accounted for to obtain any reasonable agreement.

Using Hartree–Fock (HF) and Møller–Plesset (MP) perturbation theory, Pople and co-workers explored the PELs for blocked alanine and glycine dipeptides^39^ and showed that the steric effect induced by the methyl side-chain of the alanine dipeptide had a profound effect on its conformational preferences. In a later study, Gould et al. corroborated these findings, although different capping groups and basis sets were used to model the dipeptides.^40^ Subsequently, several benchmark studies^41−44^ on the alanine dipeptide comparing different levels of theory have emerged. In a recent contribution, Fedorov and co-workers^45^ exploited a hybrid approach combining systematic scanning of the PEL and *ab initio* molecular dynamics to study the alanine dipeptide system. Interestingly, the authors identified several new minima, not characterized in earlier work, suggesting that the PELs of simple dipeptides in the gas-phase, often used as a proxy to understand the principles underlying protein folding, can exhibit unexpected features. While most of these studies have primarily focused on elucidating the thermodynamic features of the underlying landscape, Salahub and co-workers estimated the characteristic time scale of interconversion between the two prominent conformers of alanine dipeptide, namely, C_7_^eq^ and C_5_, employing *ab initio* molecular dynamics within the framework of Kohn–Sham density functional theory.^46^ They noted that the transitions between the two conformers occur on the picosecond time scale, in apparent disagreement with classical molecular dynamics simulations, in which no interconversion events were observed even after nanoseconds.

In this work, we systematically explore the PELs for two model dipeptides, namely, *N*-acetyl-*L*-alaninamide (Ac-Ala-NH_2_), and *N*-acetyl-*L*-serinamide (Ac-Ser-NH_2_), in the gas-phase using geometry-optimization-based computational tools developed within the framework of energy landscape theory.^8,47,48^ We use basin-hopping (BH) global optimization^49,50^ in conjunction with gas-phase molecular dynamics to identify low-lying minima on the PEL, and discrete path sampling (DPS)^51,52^ to locate the intervening transition states. The rearrangement pathways between the distinct peptide conformations are described geometrically in terms of interconnected minimum–transition state–minimum triples on the PEL, rendering an *a priori* choice of reaction coordinates unnecessary. Our approach is largely complementary to “rare event” sampling schemes based on explicit dynamics,^53−60^ which are routinely used to study folding surrogates, such as dipeptides, for both benchmarking and force field development.

We find that, for both Ac-Ala-NH_2_ and Ac-Ser-NH_2_, the C_7_^eq^ conformer corresponds to the free energy global minimum at 298 K, in agreement with previous experimental^29,33,61^ and theoretical studies.^39,40,43^ As anticipated by Alonso and co-workers,^29,33^ the presence of a polar side-chain in Ac-Ser-NH_2_ makes its underlying energy landscape somewhat more complex compared to Ac-Ala-NH_2_ and leads to distinct thermodynamic as well as kinetic features. The free energy gap, *ΔF*, at 298 K between the C_7_^eq^ and C_5_ conformers of Ac-Ala-NH_2_ is small (around 0.03*k*_B_*T*), explaining their coexistence, and simultaneous detection in the supersonic expansion based on their rotational signatures.^29^ On the other hand, *ΔF* ≈ 2*k*_B_*T* for Ac-Ser-NH_2_, and the C_7_^eq^ conformer dominates the equilibrium population.

## Computational Methodology

The initial structures of the capped dipeptides, Ac-Ala-NH_2_ and Ac-Ser-NH_2_, were constructed using the *tleap* module available within the AMBER14 code.^62^ The dipeptides were modeled using the AMBERff14SB force field.^63^ A preliminary exploration of the conformational space was first carried out *in vacuo* using short molecular dynamics (MD) simulations of around 2 μs. The temperature was maintained at 298 K using a Langevin thermostat,^64^ employing a collision frequency of 1.0 ps^–1^. These simulations were carried out using the AMBER14 code.^62^ Snapshots from the MD trajectories were periodically quenched to build an initial database of minima.

We then employed basin-hopping (BH) global optimization,^49,50^ as implemented within the interfaced version of the GMIN code^65^ with the AMBER9^66^ package, to locate low-lying minima that could have been missed by finite temperature MD simulations. As shown in previous work,^49,67−69^ BH has been successful in locating the putative global minima for a wide range of atomic and molecular systems characterized by landscapes with broken ergodicity. The key idea in BH is to transform the multidimensional PEL into a set of interpenetrating catchment basins, comprising the local minima. The transformation does not change the global minimum or the relative ordering of the local minima. However, the downhill barriers between the local minima in the different basins are removed.^47^ The basin transformation is coupled with steps between local minima to explore the configuration space. For each dipeptide, 100 separate BH runs with different random number seeds were carried out. Group rotation moves (rigid body rotation of a group of atoms about a predefined axis), in conjunction with random Cartesian displacements, were employed to perturb the coordinates of the current local minimum. Local minimization was carried out for each BH step using a modified version of the L-BFGS algorithm.^70^ The new minimum, thus generated, was accepted/rejected on the basis of the Metropolis criterion.

Discrete path sampling (DPS)^51,52^ was employed for further exploration of the PEL. The DPS procedure has been previously used to construct databases of stationary points (kinetic transition network) for a wide range of systems^71−77^ and provide insight into conformational transitions spanning a hierarchy of time scales. Within the DPS framework, the connectivity between different regions of the PES is described in terms of discrete paths, consisting of an interconnected sequence of local minima and intervening transition states. In accordance with the Murrell–Laidler definition,^78^ any stationary point with a single imaginary frequency (with the associated eigenvector corresponding to a reaction coordinate) is characterized as a transition state. The adjoining minima are connected to the transition state via approximate steepest-descent paths directed parallel and antiparallel to the eigenvector corresponding to the unique negative Hessian eigenvalue.

DPS runs were carried out to connect the different local minima identified from BH in a pairwise fashion. Initial guesses for the intervening transition states were obtained using the doubly-nudged^79^ elastic band^80,81^ (DNEB) method. The hybrid eigenvector-following scheme^82^ was then used to accurately converge the candidate transition state structures obtained from DNEB, until the root-mean-square (RMS) gradient fell below 10^–6^ kcal/mol Å^–1^. All the local minimizations and transition state searches were carried out using the OPTIM code^83^ interfaced to AMBER9.^66^ As the initial discrete paths found between the different pairs of minima are unlikely to be kinetically relevant, they were further refined using various sampling schemes available within the PATHSAMPLE code.^84^ Specifically, the SHORTCUT BARRIER and SHORTCUT schemes, described in previous work,^85^ were used to locate pathways characterized by lower potential energy barriers, and shorter path lengths, respectively. However, these pathway refinement schemes often lead to undersampling of some regions of the PEL, manifested in the form of artificial frustration. The UNTRAP scheme,^85^ as implemented within the PATHSAMPLE code, was used to remove this artificial frustration by systematically reconnecting selected local minima to the global minimum. This criterion for selecting minima for the connection-making attempts is based on the ratio of the potential energy barrier to the potential energy difference relative to the global minimum. The databases were expanded by sequential applications of these three refinement schemes, until no further stationary points were located. The rearrangement pathways between different dipeptide conformers were extracted from the transition network using Dijkstra’s shortest path algorithm^86^ with appropriate edge-weights.

The harmonic superposition approximation (HSA)^87,88^ was used to estimate the free energies from the stationary point databases obtained from DPS. Within the HSA, the overall canonical partition function is written as a sum of contributions from the catchment basin of each local minimum:^47^
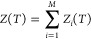
1In eq 1, *M* is the total number of minima in the stationary point database. The canonical partition function *Z*_*i*_(*T*) for local minimum *i* is expressed as^47^
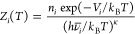
2Here, *V*_*i*_ denotes the potential energy of local minimum *i*, *n*_*i*_ is the number of distinct permutation-inversion isomers of *i*, ν̅_*i*_ is the geometric mean of the normal-mode frequencies associated with minimum *i*, and κ is the number of vibrational degrees of freedom.

The local free energy, *F*_*i*_(*T*), and the corresponding equilibrium occupation probability, *p*_*i*_^eq^(*T*), of minimum *i* are^47^
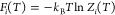
3and
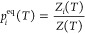
4The partition functions, and the free energies corresponding to the transition states in the stationary point database, have the same forms as those in eqs 2 and 3, except that the normal mode corresponding to the imaginary frequency is excluded from the geometric mean. The heat capacity, *C*_v_, is defined in terms of the partition function, *Z*(*T*), using standard relations from equilibrium thermodynamics:
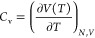
5In eq 5, *V*(*T*) is the internal energy. Using eq 2, *C*_v_ corresponding to the superposition partition function can be expressed as
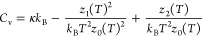
6where
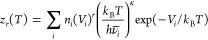
7

Harmonic transition state theory (TST) is used to compute the minimum-to-minimum rate constants:
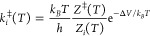
8In eq 8, *Z*^⧧^(*T*) denotes the partition function of the transition state and does not include the contribution from the normal mode with the imaginary frequency. *Z*_*i*_(*T*) is the partition function of minimum *i*, and *ΔV* is the potential energy difference between the transition state and minimum *i*. For an elementary transition from minimum *i* to minimum *j*, the total rate constant *k*_*ji*_(*T*) is obtained by summing the *k*_*i*_^⧧^(*T*) values for all transition states that connect the two minima. The overall rate constants between reactant (A) and product (B) states can be expressed in terms of these minimum-to-minimum rate constants, *k*_*ji*_, in conjunction with a Markovian assumption for the dynamics between adjacent minima. When the steady-state approximation is relaxed, we obtain the phenomenological rate constants or equivalently the mean first passage times between A and B, which describe the global dynamics. The new graph transformation (NGT) method^89,90^ was used to extract these global dynamical observables. For further details regarding the formalism, we refer the readers to the original references.^51,52,90^

The potential and free energy landscapes of the dipeptides were visualized using disconnectivity graphs.^91,92^ This representation of the landscape is simple, yet powerful, and preserves the barriers between different local minima, in contrast to low-dimensional projection-based approaches that may introduce artifacts.^93,94^

To obtain refined estimates of relative energies and barriers, the local minima corresponding to different dipeptide conformations, as well as transition states connecting them, were further reoptimized in the gas-phase at the MP2 level of theory using 6-311++G(d,p) basis sets. Previous work has shown that this level of theory is appropriate for describing the conformational preferences of small peptides.^24,33,95^ The geometry optimizations were carried out using the GAUSSIAN09 package^96^ again interfaced to the OPTIM code.^83^

## Results and Discussion

### Initial Exploration of the Conformational Space

**Figure 1:**
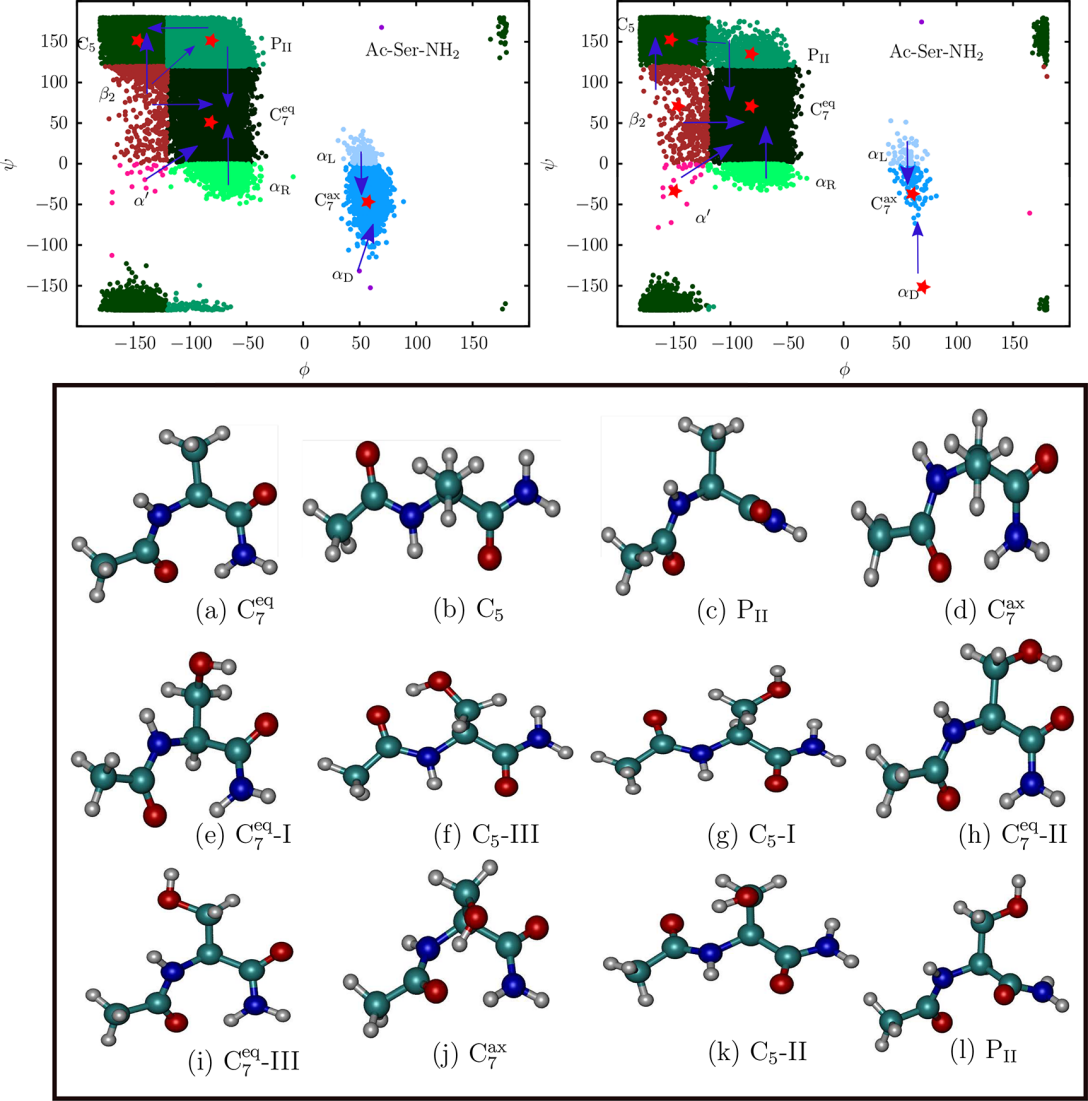
Top panel: The different conformations sampled along the gas-phase MD trajectories are shown on the Ramachandran map (left, Ac-Ala-NH_2_; right, Ac-Ser-NH_2_). The red stars denote the different potential energy basins that are identified after local minimization of snapshots from the MD trajectories at regular intervals. The blue arrows indicate the specific basins that the different conformations fall into after local minimization. The thickness of the arrows is approximately proportional to the probability of visiting a particular local minimum. The conformers are labeled according to the Ramachandran angles (ϕ, ψ), following the convention described in the study of Perez and co-workers.^97^ Bottom panel: Distinct low-energy conformers of the dipeptides. (a–d) The different conformers of Ac-Ala-NH_2_. (e–l) The different conformers of Ac-Ser-NH_2_. The β_2_, α′, and α_D_ conformers of Ac-Ser-NH_2_, which are destabilized relative to the global minimum by more than 6 kcal/mol, are not shown.

The two dipeptides can access different conformations (Figure 1 and Table S1) on the time scale of the MD simulations. The conformational space can be segregated on the basis of Ramachandran angles (ϕ, ψ), following the convention described in the study of Perez and co-workers.^97^ We find snapshots corresponding to all nine conformer families, for both the dipeptides, which suggests that our initial sampling based on MD is adequate, even if it was not exhaustive. As observed in earlier work,^97^ some of the conformers are not true geometric minima on the gas-phase PEL and relax to other geometries upon minimization. For Ac-Ala-NH_2_, there are only four potential energy minima, corresponding to the C_7_^eq^, C_5_, C_7_^ax^, and P_II_ conformer families. Although a large number of β_2_ conformers are sampled along the MD trajectory, a majority of them relax either to the C_5_ or the C_7_^eq^ form, and a minor population relaxes to the P_II_ geometry upon quenching. Most structures belonging to the P_II_ conformational cluster switch to either the C_7_^eq^ or the C_5_ form after local minimization, and only a few of them stay in the P_II_ basin. The MD simulation also samples a significant number of α_R_ conformations. However, all of them relax to the C_7_^eq^ minimum after geometry optimization (Supporting Information, Figure S1). The α′ conformer, which is adjacent to α_R_ in the Ramachandran plot, also relaxes to the C_7_^eq^ minimum upon quenching. On the other hand, all the α_L_ and α_D_ conformations relax to the C_7_^ax^ minimum.

For Ac-Ser-NH_2_, we find that, in addition to C_7_^eq^, C_5_, C_7_^ax^, and P_II_, the β_2_ and α′, and α_D_, conformers are also associated with basins of attraction on the PEL (Figure 1). However, a major population of β_2_ conformations still relax to either the C_7_^eq^ or C_5_ geometry on local minimization. A majority of the P_II_ structures also switch to C_7_^eq^ after quenching. As for Ac-Ala-NH_2_, all of the α_R_ conformations relax to C_7_^eq^, and α_L_ conformations relax to the C_7_^ax^ form after geometry optimization. Only one out of all the sampled α′ conformations quenches to the α′ basin, and the rest relax to the C_7_^eq^ geometry upon local minimization. Of the two α_D_ structures of Ac-Ser-NH_2_ that are sampled along the MD trajectory, one remains in the α_D_ geometry upon quenching, while the other switches to the C_7_^ax^ form. For both Ac-Ala-NH_2_ and Ac-Ser-NH_2_, only two snapshots out of 10^5^ adopt the α_D_ conformation. Our results suggest that, for both the dipeptides *in vacuo*, the α_D_ conformer is a high free energy minimum (if a true minimum at all), in accord with a number of previous studies employing different molecular mechanics force fields.^53,54,98^ In fact, systematic benchmarking of different *ab initio* methods^40,41,45,99^ shows that the α_D_ conformer is not a minimum on most potential energy surfaces.

To ensure that our initial sampling based on microsecond MD did not miss any relevant low-lying minimum on the PEL, we initiated BH simulations from random starting configurations of the two dipeptides. BH did not locate any new potential energy minimum, other than those already identified from the MD trajectories, suggesting that a proper exploration of the low-lying region of the underlying landscape has been achieved. The distinct low-energy conformers of Ac-Ala-NH_2_ and Ac-Ser-NH_2_ are shown in Figure 1. For both the dipeptides, the folded C_7_^eq^ conformer corresponds to the potential energy global minimum. As pointed out in earlier work,^33^ the stabilization of the C_7_^eq^ conformer can be attributed to the formation of a seven-membered ring, in which an intramolecular hydrogen-bond is formed between the acetyl carbonyl oxygen, and one of the terminal amide hydrogens. The fully extended C_5_ conformer is the second-lowest minimum for Ac-Ala-NH_2_ and is destabilized with respect to the C_7_^eq^ conformer by only ≈0.3 kcal/mol. This value is somewhat lower than that predicted by different *ab initio* methods^33,41,99,100^ and could be due to the deficiencies associated with the AMBER force field.^36,101,102^ The P_II_ minimum does not have the hydrogen-bond between the carbonyl and the amide moieties and is destabilized by around 1.1 kcal/mol relative to the C_7_^eq^ conformer. The C_7_^ax^ conformer also exhibits a seven-membered ring, much like C_7_^eq^. However, it is destabilized by around 1.6 kcal/mol because of the axial orientation of the methyl group that results in a steric repulsion between the C_β_ atom and the seven-membered ring.^45^ The relative ordering of the different conformers is in agreement with previous studies on different alanine dipeptide analogues.^41,45,99,103−105^

For Ac-Ser-NH_2_, three distinct low-energy C_7_^eq^ conformers are identified, which differ primarily in the orientation of the side-chain. Two of these conformers (C_7_^eq^-I and C_7_^eq^-II) were reported before by Alonso and co-workers.^29^ In terms of relative potential energies, C_7_^eq^-I < C_7_^eq^-II < C_7_^eq^-III. This order could be rationalized on the basis of the hydrogen-bonding interactions formed by the polar side-chain of Ser in the different conformations (Supporting Information, Figure S2). In C_7_^eq^-I, the side-chain is oriented equatorially, and it participates in two hydrogen-bonding interactions with the neighboring −N–H and −C=O groups. On the other hand, the side-chain in the C_7_^eq^-II forms a single hydrogen-bond with the −C=O group. The side-chain in C_7_^eq^-III only forms a weak hydrogen-bond with the -N–H group and is destabilized by around 3.0 kcal/mol relative to the C_7_^eq^-I conformer.

In their study, Alonso and co-workers^29^ also characterized three distinct C_5_ conformers through systematic scanning of the conformational space using semiempirical methods. These structures exhibit different interaction patterns involving the side-chain hydroxyl group (Figure 1). The local minimization of MD snapshots, as well as BH simulations, identify all these three C_5_ conformers as low-energy minima of the landscape, lying within 4.3 kcal/mol of the potential energy global minimum (the C_7_^eq^-I conformer). In fact, the C_5_-III and C_5_-I conformers are lower in potential energy compared to the other two C_7_^eq^ structures. These enhanced stabilities can also be explained in terms of the hydrogen-bonding networks formed by the polar side-chain in the two conformers (Supporting Information, Figure S2). In both structures, a six- or a seven-membered ring is formed by the interaction between the side-chain hydroxyl group, and the acetyl carbonyl oxygen (C_5_–III conformer), or one of the terminal amino hydrogens (C_5_-I conformer). No such stabilization is immediately apparent for the C_5_-II conformer, where the side-chain assumes an axial orientation. The C_7_^ax^ and the P_II_ conformers are higher in potential energy by ≈2.8 kcal/mol, and 5.3 kcal/mol, respectively, relative to the C_7_^eq^-I structure. The pronounced destabilization of the P_II_ conformer relative to its alanine counterpart can be attributed to the loss of all side-chain–backbone hydrogen-bonding interactions within this structure (Supporting Information, Figure S2).

Overall, a combination of MD and BH simulations reveal that the presence of a polar side-chain in Ac-Ser-NH_2_ significantly alters the emergent features of the PEL. It not only produces new potential energy minima, corresponding to additional conformers (β_2_, α′, and α_D_), but also increases the energy gap relative to the global minimum. In the following sections, we analyze the topological features of the free energy landscapes (FELs), as well as their emergent features, including heat capacity profiles, and rearrangement pathways among the different dipeptide conformers.

### Different Topographies of the Energy Landscapes

The potential energy minima sampled from MD and BH simulations form the initial database of stationary points. The intervening transition states between each pair of minima were identified using DPS simulations,^51,52^ and the stationary point databases were expanded using the different refinement schemes,^85^ until no new minima or transition states were found. The converged transition network for the Ac-Ala-NH_2_ dipeptide consists of 13 minima, of which the lowest six were already identified using MD and BH simulations. The other seven minima with a *cis* peptide bond are located in the high-energy region of the landscape. In addition, the network includes 102 transition states. There are 101 minima (with 57 of them exhibiting a *cis* peptide bond) and 850 transition states in the transition network for Ac-Ser-NH_2_.

**Figure 2:**
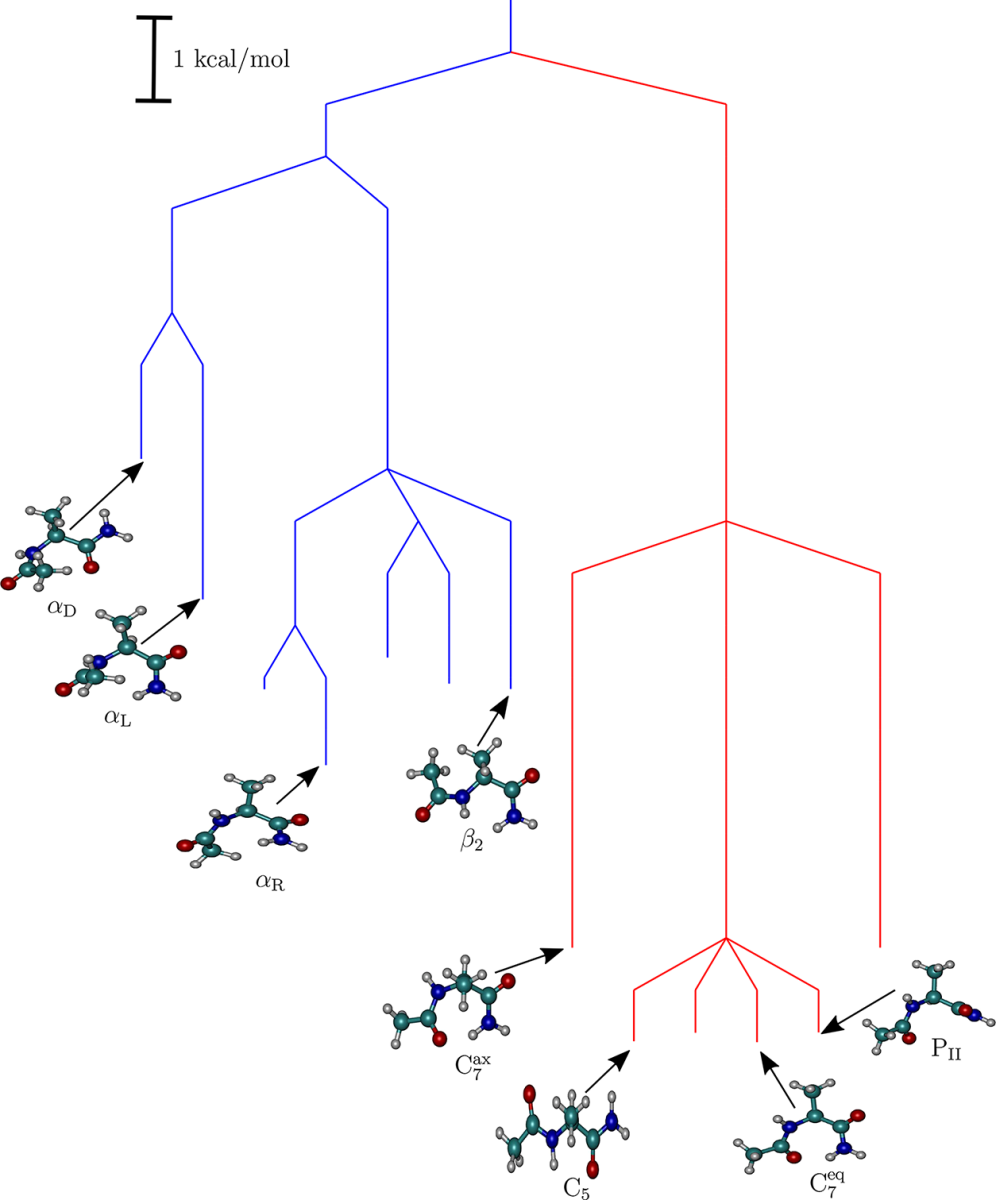
Free energy landscape for Ac-Ala-NH_2_ computed at 298 K. The branches of the disconnectivity graph are colored according to the configuration that the peptide bond adopts about the ω torsion angle. Red branches lead to minima with *trans* peptide bonds, whereas blue branches lead to minima having *cis* peptide bonds. Snapshots corresponding to the distinct dipeptide conformers are shown superimposed on the graph.

**Figure 3:**
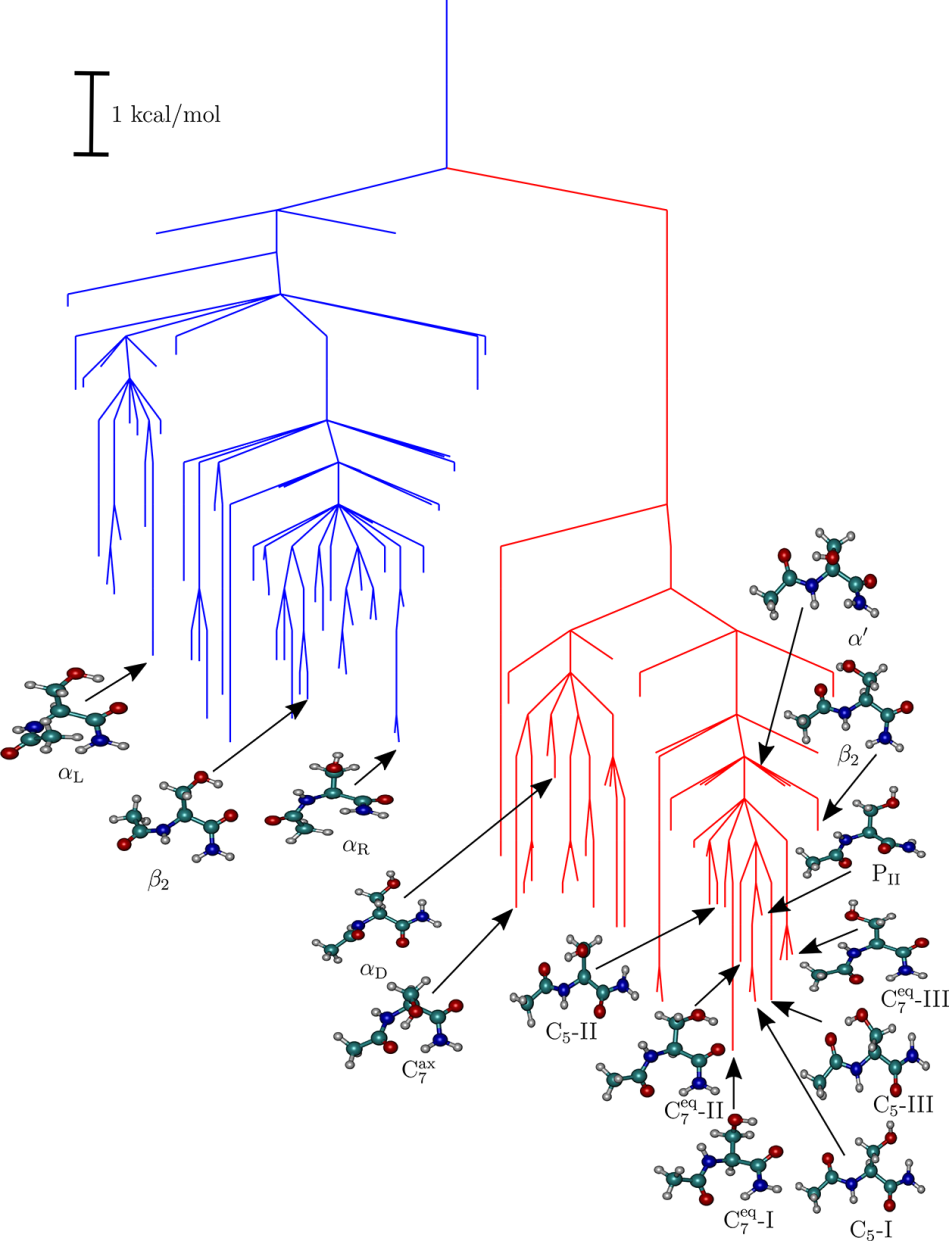
Free energy landscape of Ac-Ser-NH_2_ computed at 298 K. The coloring scheme is the same as in Figure 2. Some representative snapshots corresponding to the different dipeptide conformers are shown superimposed on the graph. It is evident from the disconnectivity graph that, in contrast to Ac-Ala-NH_2_, the conformational space of Ac-Ser-NH_2_ is quite rich.

The FELs computed at 298 K for the Ac-Ala-NH_2_ and Ac-Ser-NH_2_ dipeptides are depicted in the form of disconnectivity graphs in Figures 2 and 3, respectively. The corresponding potential energy disconnectivity graphs are included in the Supporting Information (Figures S3 and S4). The branches of the graphs are colored according to the value of the ω torsion angle, which determines whether the peptide bond assumes the *cis* or the *trans* orientation. As expected, for both the dipeptides, the biologically relevant *trans* minima occupy the low-lying regions of the energy landscape. Overall, the topographies of both FELs seem consistent with earlier IR spectra for dipeptides recorded in an argon matrix,^106^ as well as more recent rotational spectroscopy data from Alonso and co-workers.^29,33^ The free energy barrier between the *cis* and *trans* funnels is approximately 13 kcal/mol, in good agreement with previous estimates.^107^ Therefore, at physiological conditions, the *cis* configurations will not contribute significantly to the global thermodynamics, and peptide bond isomerizations will only occur at elevated temperatures.^107,108^

At 298 K, the free energy minima corresponding to the C_7_^eq^ and C_5_ conformers of Ac-Ala-NH_2_ are nearly degenerate, with a free energy difference of only around 0.02 kcal/mol. The P_II_ conformer is also stabilized further due to entropic contributions. The C_7_^ax^ conformer is destabilized relative to the C_7_ conformer by ≈1.8 kcal/mol and is separated from it by a free energy barrier of ≈7.3 kcal/mol. Our estimated values are in agreement with those previously reported in earlier studies based on different force fields and sampling techniques.^56,57,59^ We find that the *cis* funnel is not a mirror image of the *trans* funnel, neither in terms of topography, nor in the organization of different minima. The stability of the conformers within each funnel is dictated by the sterically allowed intramolecular interactions that can be formed. The overall structure of the landscape within the *cis* and *trans* funnels is also dictated by the functional form of the torsion potential within the AMBER force field, which by construction is biased toward the *trans* configuration for all non-proline amino acids. There is some evidence to suggest that a recalibration of the torsion potential could shift the conformational equilibria within the *cis* and *trans* funnels.^109^ Within the *cis* funnel, we do not find any low-lying C_7_^eq^ structure, and the α_R_ conformer corresponds to the lowest free energy minimum, followed by the β_2_, α_L_, and α_D_ conformers. Interestingly, none of the *trans* counterparts of the low-lying *cis* conformers correspond to minima on the FEL. A similar asymmetry in the organization of the conformational landscape has also been observed for peptoids,^110^ as well as model dipeptides.^45^

The C_7_^eq^-I conformer is the free energy global minimum for Ac-Ser-NH_2_, with the other C_7_^eq^ and C_5_ conformers occupying the low-energy regions of the FEL. Due to entropic stabilization, the C_5_-I conformer is nearly degenerate with C_5_-III. The free energy gap between the P_II_ and the C_7_^eq^ conformers is somewhat higher than the value obtained for the Ac-Ala-NH_2_ dipeptide, due to its enhanced enthalpic destabilization, as discussed earlier. The β_2_ and α′ conformers appear at the high free energy region of the *trans* funnel and do not contribute substantially to the equilibrium thermodynamic properties at 298 K. This observation is consistent with the relative populations obtained for these conformers from our initial microsecond MD simulations (Supporting Information, Table S1). The C_7_^ax^ conformer lies at the bottom of a competing funnel on the landscape and is separated from the C_7_^eq^ conformers by ≈7.8 kcal/mol. The α_D_ conformations lie at the top of the competing funnel, and from the organization of the FEL it is apparent that they would spontaneously relax to the C_7_^ax^ basin. Similar to Ac-Ala-NH_2_, the *cis* funnel for Ac-Ser-NH_2_ consists of low-lying conformers, such as α_L_ and α_R_, which do not have *trans* equivalents.

### Emergent Thermodynamic and Kinetic Properties

**Figure 4:**
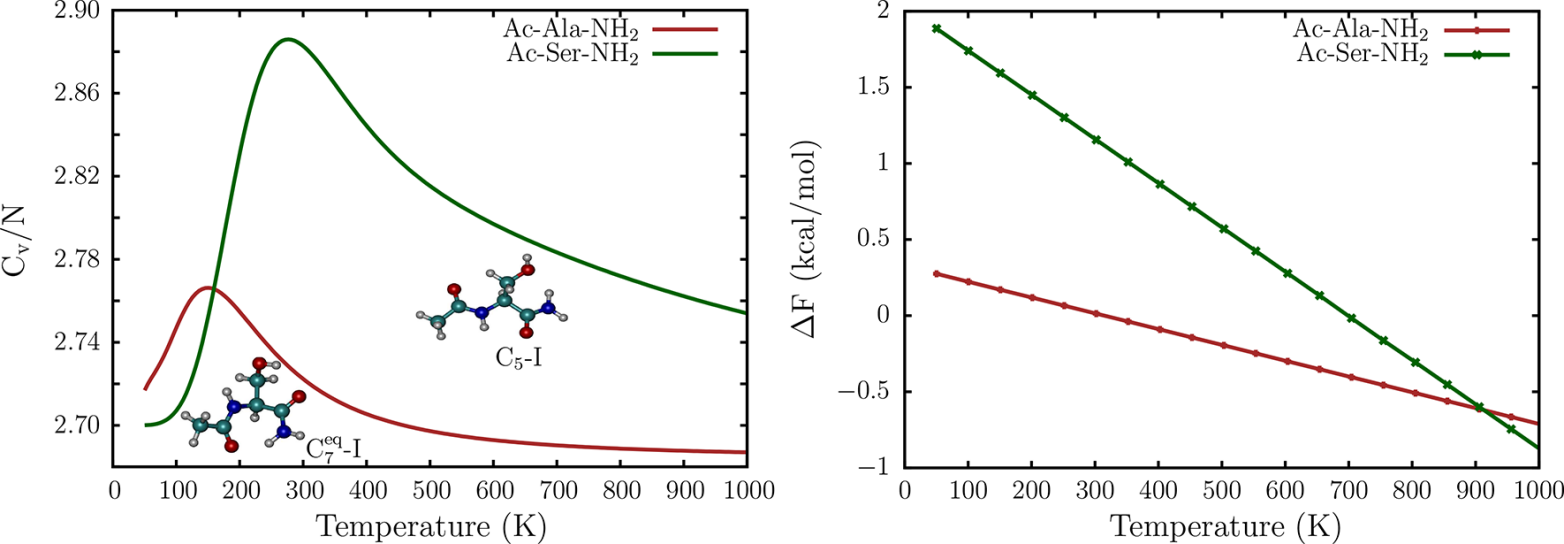
Left: The normalized heat capacity profile, *C*_v_/*N*, computed from the database of minima for Ac-Ala-NH_2_ and Ac-Ser-NH_2_. Here, *C*_v_ is scaled by the number of atoms, *N*. Right: The free energy gap, *ΔF* = *F*_C_5__ – *F*_C_7_^eq^_, between the C_7_^eq^ and the C_5_ conformers, as a function of temperature, for Ac-Ala-NH_2_ and Ac-Ser-NH_2_.

The heat capacity profiles of Ac-Ala-NH_2_ and Ac-Ser-NH_2_ provide further insight into how side-chain polarity modulates the emergent thermodynamic features of the landscape (Figure 4). For both the dipeptide sequences, there is a low-temperature peak in the heat capacity profile, which results from the competition between the low-lying C_7_^eq^ and C_5_ conformers. Similar solid–solid transitions have been characterized in detail for atomic clusters,^111^ where the interplay between enthalpy and entropy switches the free energy global minimum with temperature. The peak occurs at ≈150 K for Ac-Ala-NH_2_, in excellent agreement with previous estimates from REMD and nested sampling simulations.^55^ For Ac-Ser-NH_2_, the peak is shifted to around 278 K, reflecting the relatively larger free energy gap between the C_7_^eq^ and C_5_ conformers.

In Figure 4, we show the free energy gap, *ΔF*, between the C_7_^eq^ and the *C*_5_ conformers as a function of temperature. At low temperatures (≤150 K), which closely mimic the conditions for creating the cooled jet in experiments, *ΔF* for the Ac-Ala-NH_2_ dipeptide varies between 0.3 and 0.2 kcal/mol. These values result in a population ratio of 1.8:1 in favor of the C_7_^eq^ conformer, in precise agreement with the predictions of Alonso and co-workers.^33^ Under the same conditions, *ΔF* for Ac-Ser-NH_2_ is between 1.9 and 1.6 kcal/mol, resulting in a population ratio of C_7_^eq^:C_5_ ≥ 200:1. These contrasting thermodynamic features of the FEL explain why Ac-Ala-NH_2_ exists as mixture of C_7_^eq^ and C_5_ conformers in the gas-phase, while Ac-Ser-NH_2_ exists almost entirely in the C_7_^eq^ rotameric state.

**Figure 5:**
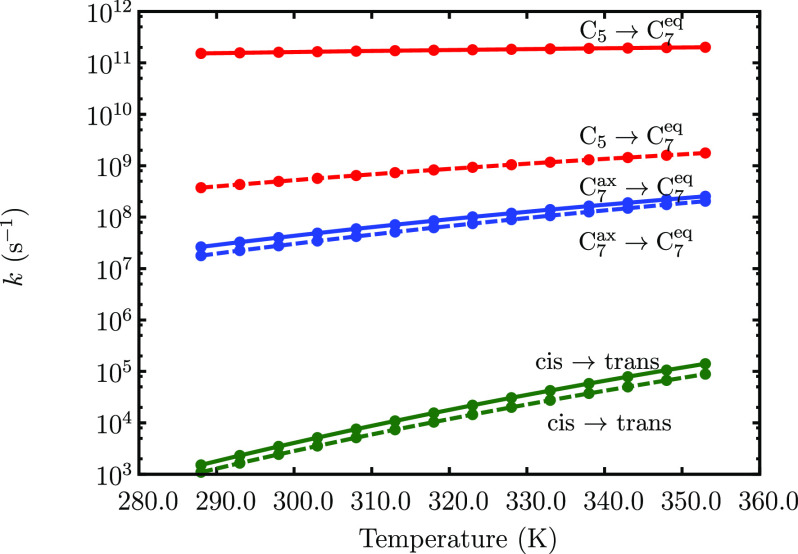
Rate constants as a function of temperature, estimated using the NGT method,^89,90^ for different conformational transitions. Solid lines denote the values for Ac-Ala-NH_2_, and the dashed lines denote the values for Ac-Ser-NH_2_.

The rate constants as a function of temperature, corresponding to different conformational rearrangements of the dipeptides, are shown in Figure 5. As expected from the topography of the FELs, the C_5_ to C_7_^eq^ transition occurs on the fastest time scales at all temperatures. We estimate that this transition occurs approximately on the picosecond time scale for Ac-Ala-NH_2_, while it is nearly 2 orders of magnitude slower in the case of Ac-Ser-NH_2_. The C_7_^ax^ to C_7_^eq^ transition occurs approximately on the microsecond time scale. Transitions involving peptide bond isomerization, from *cis* to *trans* configurations, are the slowest, with characteristic time scales of milliseconds or longer at low temperatures. In agreement with the findings of Garcia and co-workers,^108^ we observe that temperatures greater than ≈350.0 K will be required to allow bond isomerizations on the submillisecond time scale. Both the C_7_^ax^ → C_5_ and the *cis* → *trans* transition times seem to be unaffected by the presence of a polar side-chain in Ac-Ser-NH_2_. Overall, we find that, despite the inherent limitations of the harmonic superposition approach, and transition state theory, our estimated time scales are in good agreement with those predicted by explicit dynamical simulations,^57^ as well as DFT-based *ab initio* MD.^46^

### Reordering of Minima upon Reoptimization

**Figure 6:**
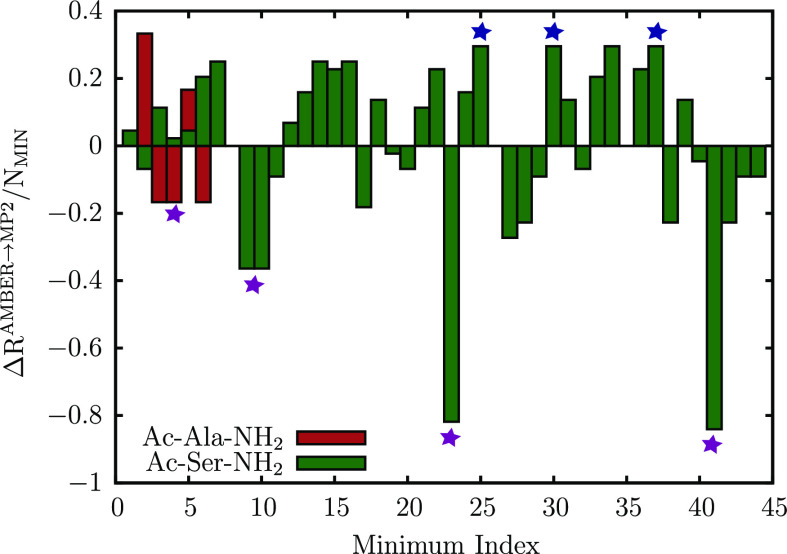
Normalized shift in ranking, *ΔR*^AMBER→MP2^/*N*_MIN_, with respect to other minima after reoptimization at the MP2/6-311++G(d,p) level. Here, *N*_MIN_ denotes the total number of minima in the stationary point database. A positive value indicates a shift toward relatively lower ranks, as the structure is destabilized relative to other minima after reoptimization, while negative values indicate that the minima are relatively stabilized and ranked more favorably after reoptimization. A purple star symbol is used to annotate those dipeptide conformations, which are maximally stabilized after reoptimization. The blue stars denote C_7_^ax^ minima of Ac-Ser-NH_2_, which are maximally destabilized after reoptimization at the MP2 level.

All minima lying within the *trans* funnel of the two dipeptides were further reoptimized at the MP2 level using a 6-311++G(d,p) basis set. The normalized shifts in the ranking of minima, *ΔR*^AMBER→MP2^/*N*_MIN_, after reoptimization are illustrated in Figure 6. Here, a negative value denotes a shift toward a higher rank, indicating that a particular minimum is stabilized relative to other minima in the database, while a positive value of *ΔR*^AMBER→MP2^/*N*_MIN_ indicates destabilization with respect to other minima. For Ac-Ala-NH_2_, the relative ordering of the various conformers in terms of potential energy is the same as the one obtained with AMBER. However, the P_II_ conformers are no longer characterized as minima on the MP2 surface, and they relax to the C_7_^eq^ geometry. These “new” C_7_^eq^ minima (denoted as purple stars in Figure 6) are lower in energy than the C_5_ minimum and hence are assigned higher ranks.

The reorganization of the minima is more elaborate for Ac-Ser-NH_2_. Although the C_7_^eq^ and C_5_ conformers still remain the lowest-energy conformers, we find that the α_D_ structures are more stabilized relative to the AMBER force field. In contrast, all of the C_7_^ax^ conformers are destabilized on the MP2 potential energy surface relative to other minima and are associated with positive values of *ΔR*^AMBER→MP2^/*N*_MIN_ ranging from 0.22 and 0.29 (Figure 6).

On the MP2 potential energy surface, we find at least two α_D_ conformers (indices 27 and 42), which are lower in energy compared to C_7_^ax^. In fact, both these minima were originally in the C_7_^ax^ form on the AMBER landscape and switched to α_D_ after reoptimization. In addition, two other C_7_^ax^ conformers (indices 13 and 24) switch to the α_L_ geometry, through major changes in the ψ torsion, and are shifted to lower ranks. The α_L_ conformer does not appear as a true geometric minimum on the AMBER potential energy surface. This discrepancy suggests that, despite the reasonable agreement with gas-phase experimental data, the AMBERff14SB force field has shortcomings, which need to be carefully evaluated in future studies. The most substantial shifts in ranking occur for two α′ conformers (indices 23 and 41, denoted as purple stars in Figure 6), which switched to the C_5_ conformation after reoptimization. The other noticeable change in ranking occurs for minimum index 9 (denoted as a purple star in Figure 6), which was originally a P_II_ minimum on the AMBER surface and switches to the C_5_ geometry upon reoptimization at the MP2 level. In fact, similar to Ac-Ala-NH_2_, the P_II_ conformers of Ac-Ser-NH_2_ generally do not exist as potential energy minima on the MP2 surface and switch to other forms, particularly C_5_.

### Rearrangement Pathways

**Figure 7:**
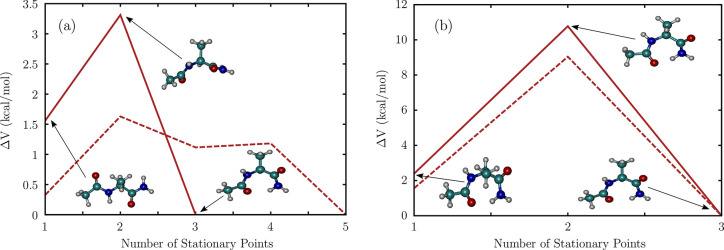
Transition paths between the different conformers of Ac-Ala-NH_2_, computed using the AMBERff14SB force field (dashed lines), and at the MP2/6-311++G(d,p) level (solid lines). (a) C_5_ to C_7_^eq^ transition. (b) C_7_^ax^ to C_7_^eq^ transition. In these profiles, *ΔV* denotes the relative potential energy with respect to the global minimum.

**Figure 8:**
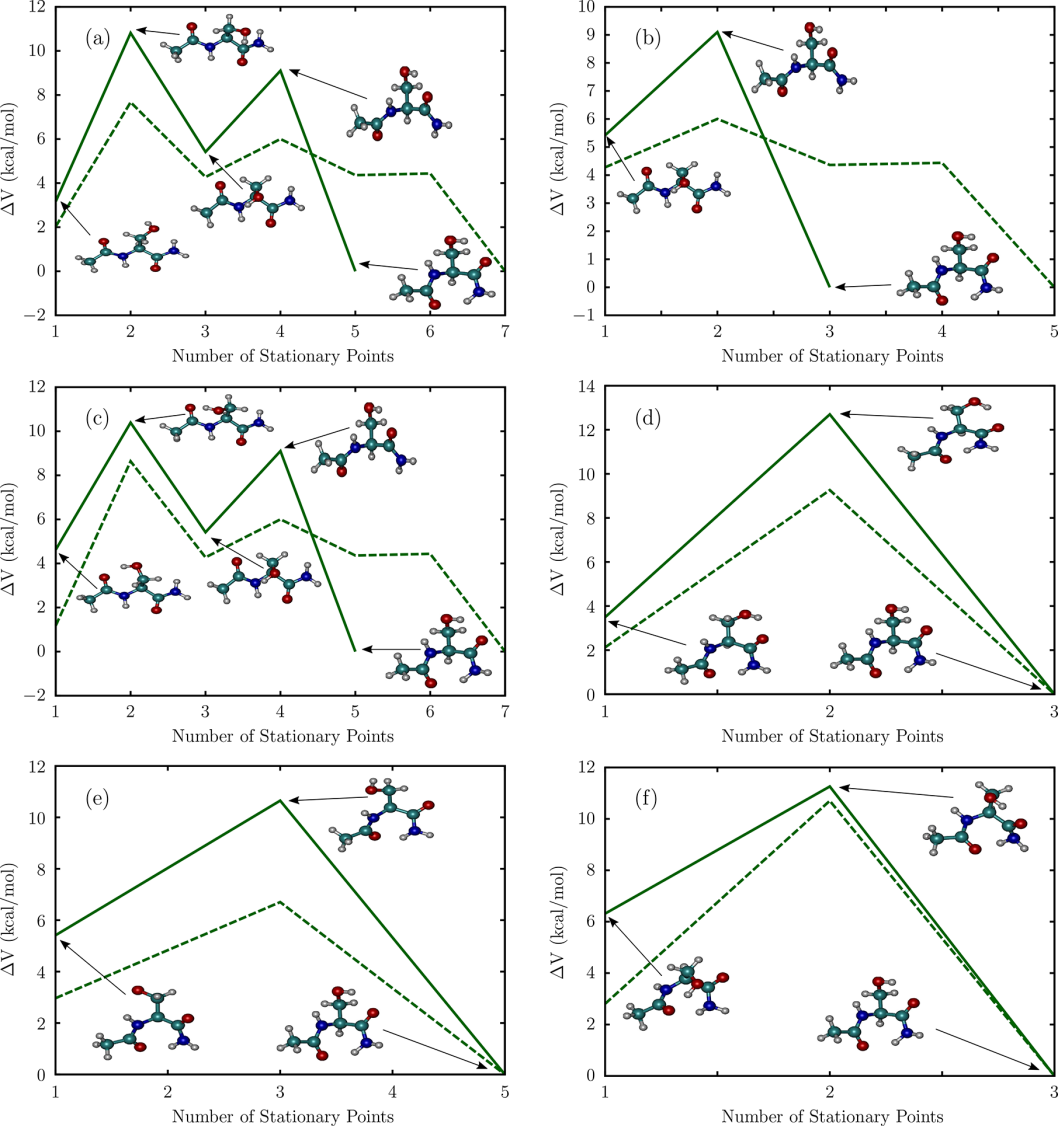
Transition paths between the different conformers of Ac-Ser-NH_2_, computed using the AMBERff14SB force field (dashed lines), and at the MP2/6-311++G(d,p) level (solid lines): (a) C_5_-I to C_7_^eq^-I, (b) C_5_-II to C_7_^eq^-I, (c) C_5_-III to C_7_^eq^-I, (d) C_7_^eq^-II to C_7_^eq^-I, (e) C_7_^eq^-III to C_7_^eq^-I, and (f) C_7_^ax^ to C_7_^eq^-I. Similar to Figure 7, *ΔV* denotes the relative potential energy with respect to the global minimum.

The rearrangement pathways between the different conformers of Ac-Ala-NH_2_, and Ac-Ser-NH_2_, were computed using the AMBER force field, as well as electronic structure calculations at the MP2 level (Figures 7 and 8). To generate connected paths on the MP2 potential energy surface, the transition states along a given rearrangement pathway (computed using AMBER) were first reoptimized at the MP2/6-311++G(d,p) level. Next, the transition state geometry was perturbed in the directions parallel and antiparallel to the reaction coordinate (eigenvector associated with the imaginary frequency) to yield the adjoining minima. Using this protocol, we are able to exactly reproduce the relative potential energies, *ΔV*_MP2_, between the different minima reported in the recent studies of Alonso and co-workers (Supporting Information, Table S2).^29,33^

The C_5_ conformer of Ac-Ala-NH_2_ switches to the C_7_^eq^ structure via a P_II_-type transition state (Figure 7a). While the rearrangement occurs in a single step on the MP2 potential energy surface, it requires two steps on the AMBER landscape. The potential energy barrier, *ΔV*, computed at the MP2 level (1.8 kcal/mol) is in excellent agreement with an early work by Schäfer and co-workers,^112^ as well as more recent estimates from *ab initio* MD.^58^ The corresponding barrier height estimated using the AMBER force field is around 1.3 kcal/mol.

The C_7_^ax^ to C_7_^eq^ rearrangement occurs in a single step (Figure 7b). Although the transition state adopts a conformation similar to the C_7_^ax^ form, its ϕ torsion angle switches from 60° to around 2°. A similar saddle point was identified along the minimum free energy path through the finite temperature string method, although the CHARMM22 force field was used to model the dipeptide.^57^ The potential energy barrier, *ΔV*, for the transition is estimated to be around 7.5 kcal/mol with the AMBER force field, and about 8.4 kcal/mol at the MP2 level. These values are consistent with previous estimates from transition path sampling,^57,59,113^ as well as milestoning.^114^

In Ac-Ser-NH_2_, the transitions from the C_5_-I and C_5_-III forms to C_7_^eq^-I occur in two steps on the MP2 potential energy surface. On the other hand, the pathways computed using the AMBER force field consist of three intervening transition states (Figure 8a,c). In contrast to the rearrangement mechanisms identified for Ac-Ala-NH_2_, none of the C_5_ → C_7_^eq^ pathways for this sequence proceed via a P_II_-type transition state. Instead, the first step in both C_5_-I → C_7_^eq^-I and C_5_-III → C_7_^eq^-I transformations involves a conformational switch to the C_5_-II minimum, through a transition state, in which the hydroxyl group of the Ser side-chain is approximately oriented between the equatorial and axial positions. The next step along the pathway on the MP2 surface involves a direct transformation to the C_7_^eq^ form, through a C_7_^eq^-like transition state, having a relatively open structure (Figure 8a−c). In the final step, the seven-membered ring in C_7_^eq^ forms in a completely downhill fashion. On the AMBER surface, there is an additional intervening step involving a rearrangement to a lower-energy C_5_-II structure, before the transition to the C_7_^eq^ form can occur (Figure 8a–c).

The transformations between the different C_7_^eq^ conformers occur in a single elementary step (Figure 8d,e). The mechanism involves disruption and reformation of the hydrogen-bond between the amide hydrogen and the acetyl carbonyl oxygen, and concomitant rotation of the Ser side-chain. The C_7_^eq^-II to C_7_^eq^-I rearrangement involves an abrupt change in the backbone configuration, as the ψ angle switches from around 50° in the C_7_^eq^-II minimum to approximately 2° in the transition state structure. For this step, we estimate a barrier height of ≈7.1 kcal/mol using AMBER, and 9.2 kcal/mol at the MP2 level. The C_7_^eq^-III → C_7_^eq^-I transition is not accompanied by such a substantial distortion of the protein backbone, and the ϕ angle changes by ≈17°. As a result, the corresponding barrier height, *ΔV*, for this transition is much less and is estimated to be 3.7 kcal/mol using the AMBER force field and around 5.2 kcal/mol at the MP2 level.

The C_7_^ax^ to C_7_^eq^-I rearrangement in Ac-Ser-NH_2_ proceeds via an α_L_ transition state (Figure 8f). Both the ψ and ϕ torsions change substantially along the transition. This transition mechanism is significantly different from the one observed for Ac-Ala-NH_2_, which proceeds through a C_7_^ax^-like transition state and involves an abrupt change only in the ϕ torsion angle. Despite these different rearrangement mechanisms, the AMBER force field results in very similar barrier heights (*ΔV* = 7.9 kcal/mol for Ac-Ser-NH_2_, and 7.5 kcal/mol for Ac-Ala-NH_2_) for the two dipeptides. This surprising equivalence rationalizes why we obtained very similar time scales for the C_7_^ax^ → C_7_^eq^ transitions at all temperatures (Figure 5). However, this equivalence of barrier heights is not supported by our MP2 calculations. *ΔV* corresponding to the C_7_^ax^ → C_7_^eq^ transition in Ac-Ser-NH_2_ is predicted to be 4.9 kcal/mol, which is approximately half of the estimated MP2 barrier height for Ac-Ala-NH_2_. This discrepancy may result from the relative destabilization of C_7_^ax^ minima on the MP2 surface.

In contrast to earlier AMBER force fields,^115^ the ff14SB variant also includes empirical corrections based on explicit solvent simulations of (Ala)_5_^63^ and has been carefully fine-tuned to reproduce NMR data,^116,117^ such as scalar *J*-couplings. The force field optimization with respect to solution-phase experiments could be responsible for the significant differences with respect to the gas-phase results obtained at the MP2 level of theory. Nonetheless, our results hint at possible deficiencies in the empirical force field and emphasize the need for more careful benchmarking in future studies.

## Conclusion

In a series of recent studies,^29,33^ Alonso and co-workers demonstrated using Fourier transform microwave spectroscopy that the model peptide Ac-Ala-NH_2_ exists as mixture of C_7_^eq^ and C_5_ conformers in the supersonic jet expansion, whereas the presence of a polar side-chain in Ac-Ser-NH_2_ conformationally locks the dipeptide in the C_7_^eq^ conformation. We trace the origin of this behavior to the contrasting features of the underlying energy landscapes. At sufficiently low temperatures, which closely mimic the experimental conditions for creating a cooled jet, the free energy gap, *ΔF*, between the C_7_^eq^ and C_5_ conformers of Ac-Ala-NH_2_ is around *k*_B_*T*, suggesting that both conformers are present in the equilibrium distribution. In contrast, for Ac-Ser-NH_2_, *ΔF* is at least 2*k*_B_*T*, and the C_7_^eq^ conformer dominates the equilibrium population.

The presence of a polar side-chain results in distinct thermodynamic, as well as kinetic, signatures. Although both dipeptides exhibit a low-temperature heat capacity peak, corresponding to the C_7_^eq^ → C_5_ conformational switch, the transition occurs at a much lower temperature in Ac-Ala-NH_2_. The rearrangements between the C_7_^eq^ and C_5_ forms also occur at least an order of magnitude faster in Ac-Ala-NH_2_. The interconversion between the C_7_^ax^ and C_7_^eq^ forms occurs on the microsecond time scale, and the dynamics seem to be independent of the sequence, despite obvious differences in the rearrangement mechanisms. However, refined calculations based on Møller–Plesset perturbation theory suggest that the apparent free energy barrier for interconversion from the C_7_^ax^ to the C_7_^eq^ form could be lowered as a result of the systematic destabilization of nearly all C_7_^ax^ minima on the MP2 surface.

There is some debate regarding the relevance of gas-phase studies on biomolecules, especially because water is ubiquitous within the cellular milieu. Besides acting as a lubricant, water molecules also significantly modulate the dynamics of the protein folding via polarization fluctuations.^118^ However, some recent reviews^119,120^ on this subject show that much can be learnt regarding the fundamental stabilizing interactions in peptides from gas-phase studies. Inside cells, proteins/peptides may interact with different environments, such as membranes and hydrophobic pockets, and deciphering the gas-phase energy landscapes is important for systematically comparing the perturbations induced by these surroundings. Many proteins even retain their native structures in solvent-free conditions, and in some cases the gas-phase structure could indeed be representative of the biologically active conformation.^121^ As pointed out by Rossi and co-workers,^122^ the gas-phase provides a “clean environment” for comparison between theory and experiment. Previous work on model dipeptides clearly suggests that an aqueous environment tends to “flatten” the free energy surface^35^ and decreases the barriers between different conformations. Some low-lying gas-phase structures may be destabilized substantially, and structures with relatively open Ramachandran angles may be favored as a result of solvent-mediated interactions. Various studies based on both *ab initio*(123) and molecular mechanics^97^ methods predict that the C_7_^eq^ conformation is not preferred in polar solvents, and the α_R_ conformation emerges as the new global minimum. In line with these previous studies, we anticipate that the relative ordering of the free energy minima for both Ac-Ala-NH_2_ and Ac-Ser-NH_2_ will change in the solution-phase, although the global topographies of the landscapes are likely to be preserved.

## Supporting Information Available

Tabulated values of the number of snapshots corresponding to each conformer sampled along the MD trajectories; figure illustrating the switch in geometry during local minimization; snapshots corresponding to the low-lying C_7_^eq^ and C_5_ conformers of Ac-Ser-NH_2_, with the hydrogen-bonding pattern explicitly shown; and potential energy disconnectivity graphs (PDF)
