# Supplementary material for: Side-Chain Polarity Modulates the Intrinsic Conformational Landscape of Model Dipeptides
Source: J Phys Chem B. 2021 May 26;125(22):5809–22. doi: 10.1021/acs.jpcb.1c02412 (PMC8279551; doi:10.1021/acs.jpcb.1c02412)
Supplement: Supplementary file 1 — jp1c02412_si_001.pdf [file jp1c02412_si_001.pdf]

Supporting Information: Side-Chain Polarity Modulates the Intrinsic Conformational Landscape of Model Dipeptides

Debayan Chakraborty,[*,†] Atreyee Banerjee,[‡,¶] and David J. Wales[*,‡]

†*Department of Chemistry, The University of Texas at Austin, 24th Street Stop A5300, Austin TX 78712, USA.*

‡*Yusuf Hamied Department of Chemistry, University of Cambridge, Lensfield Road, Cambridge CB2 1EW, UK.*

¶*Max Planck Institute for Polymer Research, 55128 Mainz, Germany*

E-mail: debayan.chakraborty@utexas.edu; dw34@cam.ac.uk

Table S1: The populations corresponding to the different dipeptide conformers sampled along the 2 μs long gas-phase MD trajectories.

Ac-Ala-NH_2		
Conformer	Number of snapshots	Population
C_7^{eq}	46176	0.46
C_5	33236	0.33
C_7^{ax}	2039	0.02
P_{II}	15824	0.16
β_2	686	0.007
α_D	3	3×10^{-5}
α_L	143	0.001
α_R	1872	0.018
α'	21	2.1×10^{-4}
Ac-Ser-NH_2		
Conformer	Number of snapshots	Population
C_7^{eq}	77285	0.78
C_5	19901	0.19
C_7^{ax}	90	9×10^{-4}
P_{II}	1269	0.012
β_2	462	4.6×10^{-3}
α_D	2	2×10^{-5}
α_L	54	5.4×10^{-4}
α_R	914	9.1×10^{-3}
α'	23	2.3×10^{-4}

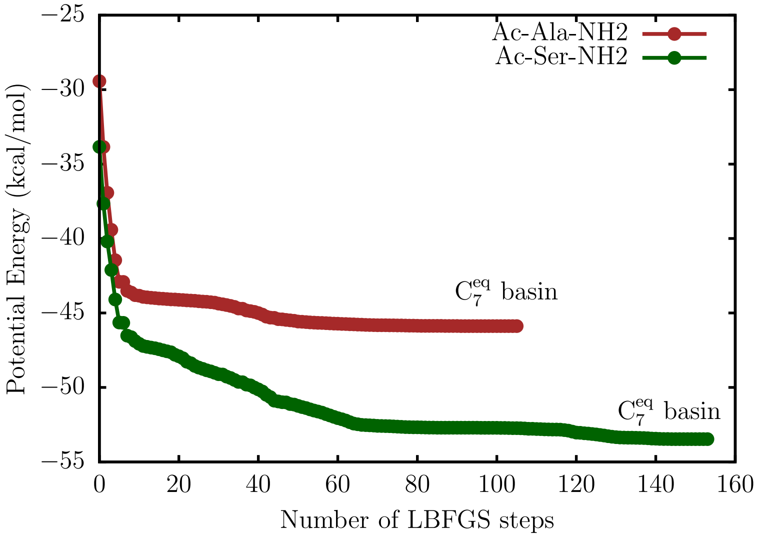

Figure S1. An illustration of the decrease in the potential energy of a structure, sampled along the gas-phase MD trajectory, during local minimization using the L-BFGS method. Here, we show how a structure originally in the α_R conformation switches to the C_7^{eq} form upon local minimization.

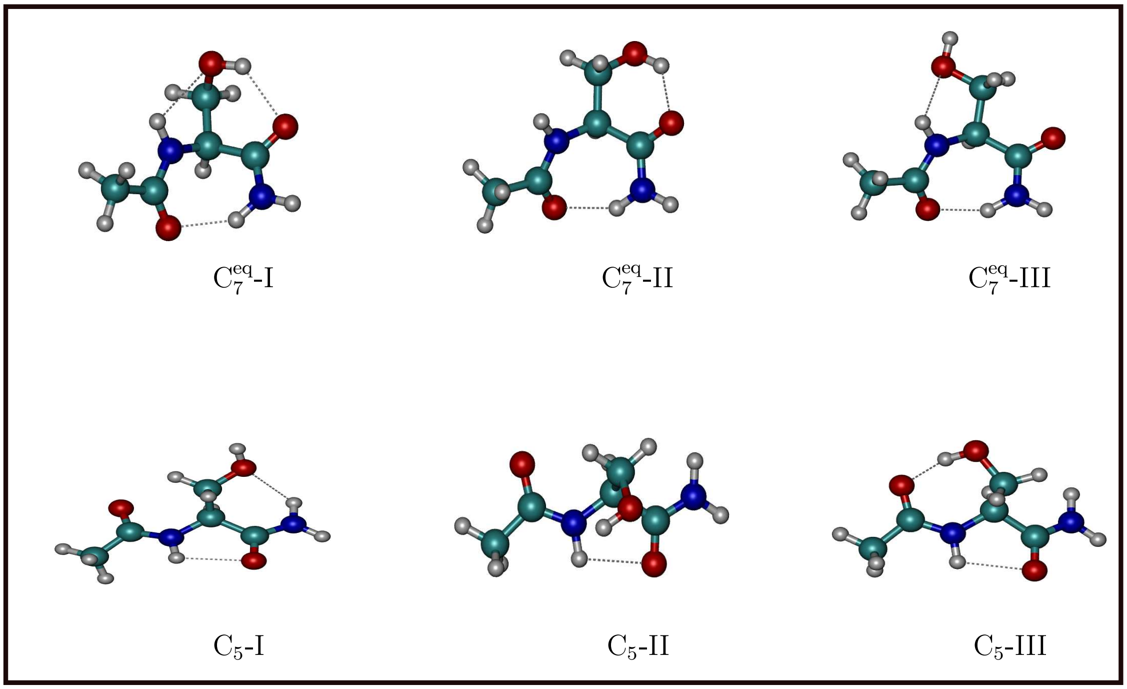

Figure S2. Snapshots corresponding to the low-lying C_7^{eq} and C_5 conformers of Ac-Ser-NH_2. The hydrogen-bonding interactions (grey dotted lines) are explicitly shown. Top panel: In the C_7^{eq}-I structure (potential energy global minimum) there is one hydrogen-bonding interaction between one of the terminal amino hydrogens, and the acetyl carbonyl oxygen, and two hydrogen-bonding interactions involving the Ser side-chain. In both the C_7^{eq}-II and C_7^{eq}-III conformers, there is only one backbone-side-chain hydrogen-bonding interaction. Bottom panel: The C_5-I and C_5-III conformers consist of hydrogen-bonding interactions between the backbone and the side-chain. In contrast, there no such interaction in C_5-II.

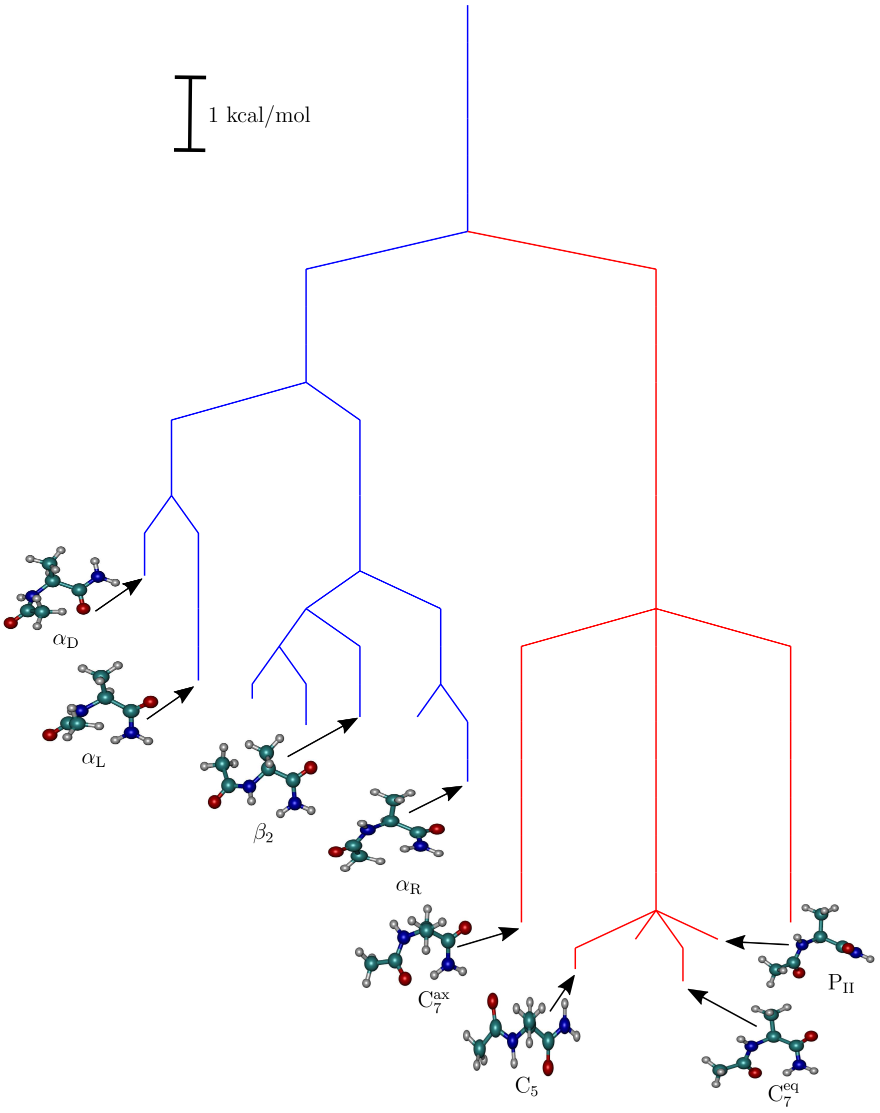

Figure S3. The potential energy landscape for Ac-Ala-NH_2 shown in the form of a disconnectivity graph. The coloring of the branches follows the same scheme as in Figure 2. Snapshots corresponding to the distinct dipeptide conformers are also shown superimposed on the graph.

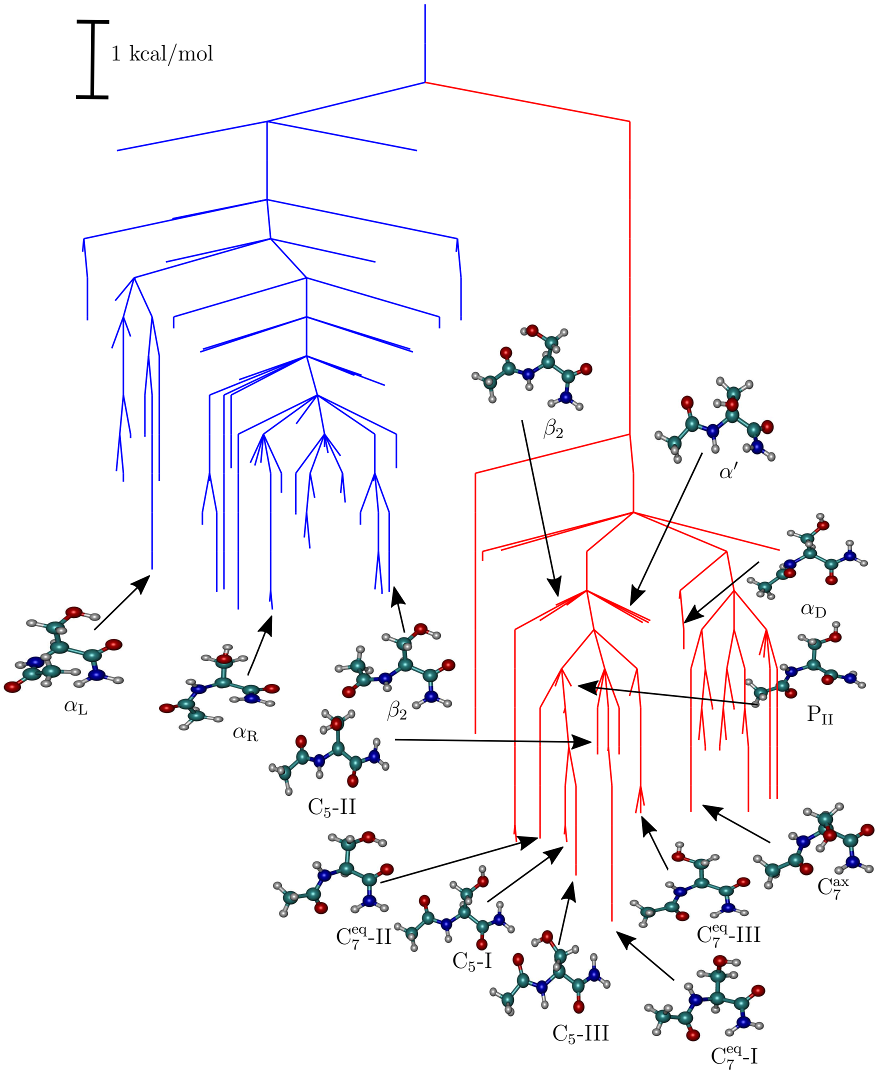

Figure S4. The potential energy landscape for Ac-Ser-NH_2 shown in the form of a disconnectivity graph. The color of the branches follows the same scheme as in Figure 2. Some representative snapshots corresponding to distinct dipeptide conformers are also shown superimposed on the graph.

Table S2: A comparison of the relative potential energies for the different dipeptide minima estimated at the MP2 level with reference data.[a,d] Values reported by Alonso and coworkers at the MP2/6-311++G(d,p) level.[1,2] [b] Values reported Freisner and coworkers[3] at the MP2/cc-PVTZ/MP2/6-31G(d) level.[c] Values reported by Dixon and coworkers[4] at the MP2/aug-ccPVQZ/MP2-aug-ccPVDZ level.

Ac-Ala-NH_2		
Conformer	ΔV_{MP2} (kcal/mol)	ΔV_{MP2}^{ref} (kcal/mol)
C_7^{eq}	0	$0^{a,b,c}$
C_5	1.6	$1.6^{a,b,c}$
C_7^{ax}	2.4	$2.4^{a,b,c}$
Ac-Ser-NH_2		
C_7^{eq}-I	0	0^{d}
C_7^{eq}-II	3.5	3.5^{d}
C_7^{eq}-III	5.4	–
C_5-I	3.2	3.2^{d}
C_5-II	5.4	5.4^{d}
C_5-III	4.6	4.6^{d}
C_7^{ax}	6.3	–

References

(1) Cabezas, C.; Varela, M.; Cortijo, V.; Jiminez, A. I.; Pena, I.; Daly, A. M.; Lopez, J. C.; Cativela, C.; Alonso, J. L. The alanine model dipeptide Ac-Ala-NH2 exists as a mixture of Ceq7 and C5 conformers. *Phys. Chem. Chem. Phys.* **2013**, *15*, 2580–2585.

(2) Cabezas, C.; Robben, M. A. T.; Rijs, A. M.; Pena, I.; Alonso, J. L. Fourier transform microwave spectroscopy of Ac-Ser-NH2: the role of side chain interactions in peptide folding. *Phys. Chem. Chem. Phys.* **2015**, *17*, 20274–20280.

(3) Beachy, M. D.; Chasman, D.; Murphy, R. B.; Halgren, T. A.; Friesner, R. A. Accurate ab Initio Quantum Chemical Determination of the Relative Energetics of Peptide Conformations and Assessment of Empirical Force Fields. *J. Am. Chem. Soc.* **1997**, *119*, 5908–5920.

(4) Vargas, R.; Garza, J.; Hay, B. P.; Dixon, D. A. Conformational Study of the Alanine Dipeptide at the MP2 and DFT Levels. *J. Phys. Chem. A* **2002**, *106*, 3213–3218.
